# Comparative genomics of koala, cattle and sheep strains of *Chlamydia pecorum*

**DOI:** 10.1186/1471-2164-15-667

**Published:** 2014-08-08

**Authors:** Nathan L Bachmann, Tamieka A Fraser, Claire Bertelli, Martina Jelocnik, Amber Gillett, Oliver Funnell, Cheyne Flanagan, Garry S A Myers, Peter Timms, Adam Polkinghorne

**Affiliations:** Faculty of Science, Health, Education and Engineering, University of the Sunshine Coast, Sippy Downs 4558, Queensland, Australia; Institute for Health and Biomedical Innovation, Queensland University of Technology, Kelvin Grove 4059, Queensland, Australia; SIB Swiss Institute of Bioinformatics, Lausanne, Switzerland; Australia Zoo Wildlife Hospital, Beerwah, Queensland 4519 Australia; Adelaide Hills Animal Hospital, Stirling, South Australia 5152 Australia; Port Macquarie Koala Hospital, Port Macquarie, NSW 2444 Australia; Institute for Genome Sciences, University of Maryland School of Medicine, Baltimore, 21201 USA; Center for Research on Intracellular Bacteria, Institute of Microbiology, University Hospital Center and University of Lausanne, Lausanne, Switzerland

**Keywords:** *Chlamydia pecorum*, Single nucleotide polymorphism, Pseudogene, Cytotoxin

## Abstract

**Background:**

*Chlamydia pecorum* is an important pathogen of domesticated livestock including sheep, cattle and pigs. This pathogen is also a key factor in the decline of the koala in Australia. We sequenced the genomes of three koala *C. pecorum* strains, isolated from the urogenital tracts and conjunctiva of diseased koalas. The genome of the *C. pecorum* VR629 (IPA) strain, isolated from a sheep with polyarthritis, was also sequenced.

**Results:**

Comparisons of the draft *C. pecorum* genomes against the complete genomes of livestock *C. pecorum* isolates revealed that these strains have a conserved gene content and order, sharing a nucleotide sequence similarity > 98%. Single nucleotide polymorphisms (SNPs) appear to be key factors in understanding the adaptive process. Two regions of the chromosome were found to be accumulating a large number of SNPs within the koala strains. These regions include the *Chlamydia* plasticity zone, which contains two cytotoxin genes (*tox*A and *tox*B), and a 77 kbp region that codes for putative type III effector proteins. In one koala strain (MC/MarsBar), the *tox*B gene was truncated by a premature stop codon but is full-length in IPTaLE and DBDeUG. Another five pseudogenes were also identified, two unique to the urogenital strains *C. pecorum* MC/MarsBar and *C. pecorum* DBDeUG, respectively, while three were unique to the koala *C. pecorum* conjunctival isolate IPTaLE. An examination of the distribution of these pseudogenes in *C. pecorum* strains from a variety of koala populations, alongside a number of sheep and cattle *C. pecorum* positive samples from Australian livestock, confirmed the presence of four predicted pseudogenes in koala *C. pecorum* clinical samples. Consistent with our genomics analyses, none of these pseudogenes were observed in the livestock *C. pecorum* samples examined. Interestingly, three SNPs resulting in pseudogenes identified in the IPTaLE isolate were not found in any other *C. pecorum* strain analysed, raising questions over the origin of these point mutations.

**Conclusions:**

The genomic data revealed that variation between *C. pecorum* strains were mainly due to the accumulation of SNPs, some of which cause gene inactivation. The identification of these genetic differences will provide the basis for further studies to understand the biology and evolution of this important animal pathogen.

**Electronic supplementary material:**

The online version of this article (doi:10.1186/1471-2164-15-667) contains supplementary material, which is available to authorized users.

## Background

*Chlamydia* are widely distributed and highly successful bacterial pathogens that only replicate inside eukaryotic cells, which is a key factor in their ability to remain hidden from the host immune response and to cause persistent infections [1]. Seemingly contrary to this highly adapted intracellular lifestyle, the majority of the eleven currently described species in the genus *Chlamydi*a, can infect multiple host species. The best example of this is *Chlamydia psittaci*, primarily recognised as an avian pathogen, but is also known to infect and cause disease in cattle, sheep, pigs and horses, while posing a zoonotic risk for humans [2]. Other species such as *C. pecorum*, *C. abortus* and *C. pneumonia*e, can infect multiple hosts as well [3–5]. For each of these species, there is little understanding of the mechanisms involved in the adaptation to different niches, especially considering that different strains within a species share nearly identical genomes with a DNA sequence similarity of > 98% [6, 7].

The adaptation of bacterial pathogens to specific niches is driven by the evolutionary “arms race” that takes place between the host and the bacterium [8]. The host’s immune system can provide a selective pressure for the accumulation of mutations in the genes of the bacterium. Although the majority of polymorphisms within genes cause synonymous changes, which are indicative of purifying selection for the encoded protein to maintain its current function and structure, a small number of genes will accumulate non-synonymous substitutions that result in protein variation [9]. Recent data analysing the accumulation of these single nucleotide polymorphisms (SNPs) in *C. trachomatis* has emphasized that this will be key to understanding the host adaptation of each the chlamydial species [10].

An interesting example of chlamydial pathogenicity and intra-species host adaptation potentially lies in an analysis of the animal pathogen, *C. pecorum. C. pecorum* is a widespread pathogen of economically important livestock species such as cattle, sheep, goats and pigs. In livestock, infections of *C. pecorum* manifest as a range of diseases such as polyarthritis, pneumonia, conjunctivitis and encephalomyelitis, while also being linked to diseases of the gastrointestinal and urogenital tracts [11, 12]. Beyond these reports, perhaps the most common outcome of *C. pecorum* infection is the absence of disease symptoms [13]. However, even in animals that are asymptomatic, there is evidence for a subclinical pathological effect [14]. While *C. pecorum* infections in livestock are of economic concern to primary producers globally, the best example of the pathogenic potential of this obligate intracellular bacterium is through the ongoing association between *C. pecorum* infection of the koala, a native Australian marsupial, and debilitating ocular and urogenital tract diseases [15, 16]. In this capacity, *C. pecorum* is a key threatening process to the long-term survival of this native species [17].

In the absence of genome sequence data for the representative strains infecting each host species, efforts to understand the genetic relationship and host adaptation of *C. pecorum* strains infecting livestock and koalas has centered on the use of molecular typing methods. A previous study that investigated the molecular epidemiology of *C. pecorum* using a Multi Locus Sequence Analysis (MLSA) typing scheme on both livestock and koala *C. pecorum* strains suggested that there is limited genetic diversity between strains and that they share a common ancestor [18]. This finding was in contrast to previous reports utilising an alternative molecular target, *omp*A, encoding the *Chlamydia* major outer membrane protein, a porin responsible for nutrient transfers, attachment and structural support [19]. However, the *omp*A gene is likely to be evolving at a faster rate than the rest of the genome since it is located on the cell surface and, as a result, the gene is under high positive selection [20]. More recently, three *C. pecorum* strains from ruminant animals were sequenced revealing a high level of sequence similarity and gene content [21]. In this setting, it is vital to employ additional whole genome sequencing of non-ruminant *C. pecorum* strains in order to fully understand the genetic diversity between *C. pecorum* strains and to identify genes that could be involved in adaptation to different hosts.

In this study, the genome sequences of *C. pecorum* strains isolated from three koalas and a sheep were compared to identify genes that could play a potential role in adaptation to different hosts and to gain insight into genetic diversity and evolution. In addition, the koala *C. pecorum* genomes were compared against each other in order to examine the genetic diversity between conjunctival and urogenital *C. pecorum* strains. The survey of the broader diversity of selected regions of the *C. pecorum* genome in a range of clinical samples from Australian livestock and koalas was also conducted.

## Results

### Phylogenetic relationship of *C. pecorum*as revealed by whole genome sequencing

The draft genomes of three *C. pecorum* strains isolated from koalas with clinical symptoms (*C. pecorum* IPTaLE, *C. pecorum* DBDeUG and *C. pecorum* MC/MarsBar) each comprise a single scaffold of approximately 1.1 Mbp (Table 1). The chromosomes have a GC content of 41% and an average read coverage of 1500×. The fourth draft genome was from the *C. pecorum* VR629 (IPA) strain that was isolated from the joint fluid of a sheep suffering from polyarthritis in the USA. None of the strains sequenced possessed the cryptic chlamydial plasmid. The three koala *C. pecorum* strains have been typed based on the *omp*A gene and each strain represents a different *omp*A serotype (Table 1) [22].

**Table 1:** ***C. pecorum*** **draft genomes sequenced in this study**

	***C. pecorum***IPTaLE	***C. pecorum***DBDeUG	***C. pecorum***MC/MarsBar	***C. pecorum***VR629
**Genotype**	A	F	G	n/a
**Source**	Koala	Koala	Koala	Sheep
**Year of Isolation**	2010	2010	2009	1968
**Country**	Australia	Australia	Australia	USA
**Isolation tissue**	Eye	UGT	UGT	Joint
**Disease**	Conjunctivitis	UTI	Chronic cystitis	Polyarthritis
**Total number of reads**	17011050	13889239	15011176	16587782
**Number of contigs**	14	8	14	5
**N50**	277726	587274	575200	478577
**Assembly length (bp)**	1,090,201	1,092,392	1,090,698	1,104,572
**Number of CDS**	990	985	980	971
**% GC content**	41	41	41	41

**Figure 1:**
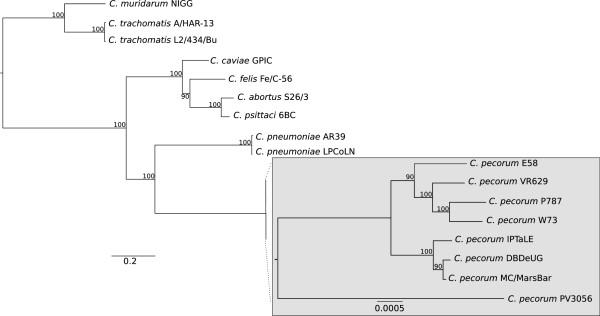
**Phylogeny of** ***Chlamydia*** **species.** The maximum-likelihood tree was reconstructed using PhyML with the GTR substitution model based on the nucleotide sequence of 152 orthologous genes. Bootstrap values are shown as percentages of 500 replicates. The scale bars represent the number of substitutions per site. The gray box shows the within-species phylogeny of the sequenced *C. pecorum* strains and is magnified.

To determine the relationship between the four sequenced *C. pecorum* strains, a phylogenetic tree was constructed based on the sequence of 152 conserved genes across eight *Chlamydia* species. The inferred topology of the phylogenetic tree (Figure 1) is consistent with other phylogenetic analysis of *Chlamydia* species [23]. The phylogenetic reconstruction indicates that the closest relative of *C. pecorum* is *C. pneumoniae*. The phylogenetic analysis revealed that the three koala *C. pecorum* strains are part of a separate lineage from the ruminant *C. pecorum* strains sequenced in this study and those previously described [21, 24]. The two urogenital *C. pecorum* strains (MC/MarsBar and DBDeUG) grouped more closely together, away from the conjunctival *C. pecorum* IPTaLE isolate.

### Whole genome comparison revealed a high level of gene conservation between *C. pecorum*strains from koalas and livestock

Whole genome comparisons confirmed that the four draft *C. pecorum* genomes included in this study are highly conserved and syntenic, with similar gene content. Comparisons against the genomes of other *Chlamydia* species revealed that *C. pecorum* contains several regions that differ significantly to other *Chlamydia* species (see Additional file 1). The variable regions include the chlamydial plasticity zone (PZ), a genomic island located near the terminus of replication and a cluster of genes that encode polymorphic membrane proteins (PMPs). Like the other *C. pecorum* genomes studied to date [21], the four draft *C. pecorum* genomes sequenced encode a near intact tryptophan biosynthesis operon but they are missing the *trpE/G* genes. The genomes of the *C. pecorum* strains are highly conserved with a DNA sequence identity ranging from 98.5 to 98.8%. The conservation of gene content and sequence similarity between *C. pecorum* strains is consistent with other *Chlamydia* species [6, 7].

### Single nucleotide polymorphisms contribute to the genetic diversity between the *C. pecorum*genomes

**Figure 2:**
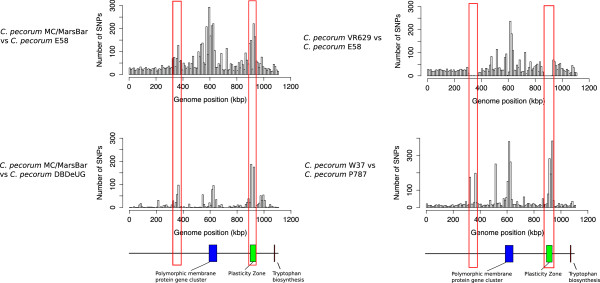
**Distribution of SNPs in** ***C. pecorum*** **genomes.** The histograms show the number of SNPs in relation to the genomic positions between different *C. pecorum* genomes with a window size of 10 kb. The top left hand graph shows the SNP distribution between the koala *C. pecorum* MC/MarsBar genome and the livestock *C. pecorum* E58 genome while the bottom left hand graph shows the number of SNPs between two koala *C. pecorum* strains (MC/MarsBar and DBDeUG). The right hand graphs display the distribution of SNPs observed between the two USA livestock *C. pecorum* strains (VR629 and E58) and two of the European livestock strains (W37 and P787). The red boxes mark the positions of the two SNP hotspot regions identified in the koala *C. pecorum* strains. The first polymorphic region starts at 300 kb and is approximately 77 kb in length and the second region includes the plasticity zone.

**Figure 3:**
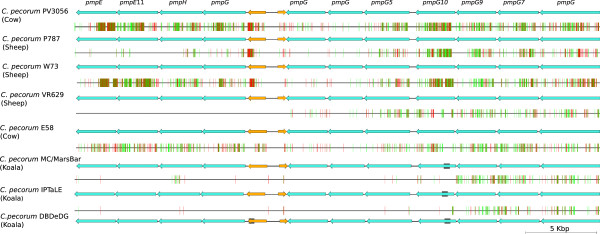
**Visual representation of the cluster of PMP genes in** ***C. pecorum*** **genome.** The blue arrows represent the PMPs and the orange arrows represent hypothetical proteins. The vertical lines mark the location and type of SNPs (green for synonymous and red for non-synonymous). The gaps in the genome assembly are marked by the gray rectangles and sizes of these gaps were estimated using pair-end read data. The base coordinates of this region in *C. pecorum* E58 are 590994..627950. The image was generated using Easyfig [25].

Although the overall gene content is conserved between each *C. pecorum* genome, there are a significant number of SNPs that contribute to variation between the *C. pecorum* strains*.* The number of predicted SNPs between livestock and koala *C. pecorum* strains ranges from 4914 to 6438 SNPs. Between the livestock *C. pecorum* strains the number of polymorphisms range from 3533 to 4129 SNPs with the exception of the phylogenetically distinct *C. pecorum* PV3056 strain which differs from the other livestock strains by 15 077 SNPs. Comparisons of the three koala *C. pecorum* genomes revealed that there are 1461-1747 SNPs (Table 2). The distribution of SNPs was plotted across the whole genome using a custom R script to reveal regions that are accumulating mutations (Figure 2). The region with the largest number of SNPs in the *C. pecorum* genomes is a 36 kb gene cluster encoding 11 PMPs. Between *C. pecorum* E58 and *C. pecorum* VR629, there are 271 synonymous and 220 non-synonymous SNPs within the PMP cluster, with the majority of SNPs found in the *pmp*G subfamily (Figure 3). Between the livestock *C. pecorum* E58 and koala *C. pecorum* MC/MarsBar isolates, this *pmp* cluster contains 461 synonymous and 433 non-synonymous SNPs making this gene cluster the most variable region between *C. pecorum* strains from different hosts. Comparison between the genomes of koala *C. pecorum* strains indicates that most of the variation observed in this region occurs with the *pmp*G genes. The adaptation to different hosts is likely the result of small and subtle changes that occurred in the genomes rather than differences in the gene content.

**Table 2:** **Total SNPs in** ***C. pecorum*** **genomes using** ***C. pecorum*** **MC/MarsBar as a reference**

Strain	Host	No. of SNPs within genes	No. of SNPs in intergenic regions	Total no. of SNPs
*C. pecorum* E58	Cattle	5454	483	5937
*C. pecorum* PV3056	Cattle	5869	569	6438
*C. pecorum* W73	Sheep	4526	430	4956
*C. pecorum* P787	Sheep	4534	380	4914
*C. pecorum* VR629	Sheep	4704	302	5006
*C. pecorum* IPTaLE	Koala	1619	128	1747
*C. pecorum* DBDeUG	Koala	1357	104	1461

### SNPs within koala *C. pecorum*strains highlight genes potentially associated with host adaptation to marsupials

The distribution of SNPs across the *C. pecorum* genomes revealed that there are two regions in the koala and European livestock *C. pecorum* strains that are hotspots for the accumulation of SNPs (Figure 2). The corresponding highly polymorphic regions do not show a similar level of diversity between the sheep and cattle *C. pecorum* strains isolated from USA. The first region, located near the origin of replication, is approximately 77 kbp in length. A detailed analysis of this region revealed the presence of genes that encode for putative type III effector proteins, chaperones and other potential virulence-related proteins. Also found here is a cluster of genes that encode the inner membrane components of the Type III secretion system (T3SS). In contrast to many other bacterial pathogens where the genes for the T3SS apparatus are clustered together on pathogenicity islands, *Chlamydia* T3SS genes are located in four separate clusters disseminated throughout the genome [26, 27]. In addition, there is a sulfur transfer system encoded by the *suf*BCD operon and *suf*S similar to a system in *E. coli;* the rest of the genes located within this region are predicted to be involved in various metabolic processes [28].

**Figure 4:**
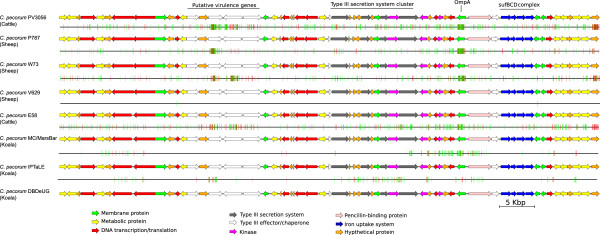
**Visual representation of the 77 kbp SNP hot-spot region.** The arrows represent genes and are coloured according to predicted functions inferred by BLAST searches. The vertical lines mark the location of SNPs with the green lines indicating synonymous SNPs and the red lines indicating non-synonymous SNPs. This region includes the *sufBCD* operon, which encodes an iron uptake system and a gene cluster that encodes the inner membrane components of the Type III secretion system. Also encoded in this region are several putative Type III effectors and chaperones. The base coordinates of this region in *C. pecorum* E58 are 306156..383066. The image was created with Easyfig [25].

The 77 kbp region is a hotspot for SNPs in both the Australian koala strains (*C. pecorum* MC/Marsbar, *C. pecorum* IPTaLE and *C. pecorum* DBDeUG) and European livestock strains (*C. pecorum* PV3056, *C. pecorum* W37 and *C. pecorum* P787) but this region is conserved between the two USA livestock strains (*C. pecorum* VR629 and *C. pecorum* E58) (Figure 4). Between *C. pecorum* E58 and *C. pecorum* MC/MarsBar, we observed 307 synonymous and 208 non-synonymous SNPs located within genes in the 77 kbp region. The majority of the non-synonymous SNPs are associated with putative virulence factors, including the Type III effector proteins. A selection analysis was carried out on each gene within this region between *C. pecorum* E58 and *C. pecorum* MC/MarsBar to determine if any genes are under positive selection [29]. Between the livestock and koala strains, most of the genes in this region are under purifying selection with a d_n_/d_s_ ratio of less than 1. However, we observed three genes that are under positive selection, including one of the putative Type III effectors (locus tags: CpecG_0280, CpecA_0283, CpecF_0282) and two conserved hypothetical proteins (see Additional file 2). Within the koala *C. pecorum* strains themselves, all the genes in this region are under purifying selection with mostly synonymous SNP differences, with the exception of the *srp*A1 gene (locus tags: CpecG_0278, CpecA_0281, CpeF_0280). The SrpA1 protein has 100% sequence identity between *C. pecorum* MC/MarsBar and *C. pecorum* IPTaLE, however it only shares 85% sequence identity to the SrpA1 homolog from *C. pecorum* DBDeUG (see Additional file 3). The C-terminal domain of SrpA1 was conserved across all the *C. pecorum* genomes and it is the N-terminal domain that is variable even between *C. pecorum* koala strains.

**Figure 5:**
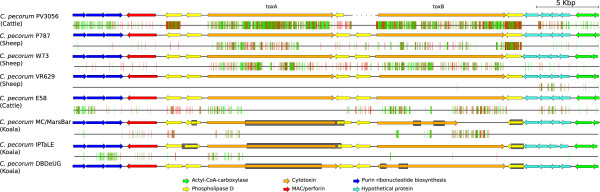
**Visual representation of the** ***C. pecorum*** **plasticity zone.** The arrows represent genes and they are colored according to function, which is illustrated in the legend. The vertical lines mark the location and type of SNPs (green for synonymous and red for non-synonymous). The gaps in the genome assembly are marked by the gray rectangles and sizes of these gaps were estimated using pair-end read data. The base coordinates of the plasticity zone in *C. pecorum* E58 are 897456..939618. The image was generated using Easyfig [25].

The second genomic region with a high frequency of SNPs is the PZ (Figure 5). However, it should be noted that in the koala *C. pecorum* genomes, there are assembly gaps within the PZ and that the number of predicted SNPs in the PZ is likely an under estimation. Paired-end data is used to estimate the size of the gaps, which suggest that the gene content of the PZ is identical in each of the genomes. In *C. pecorum*, the PZ is 42 kb with the acetyl-CoA-carboxylases genes (*acc*B and *acc*C) located at the 5′ boundary and the *gua*AB and *add* genes at the 3′ end [21]. The PZ in koala *C. pecorum* strains encodes two cytotoxin genes in tandem, in this study designated as *tox*A and *tox*B*.* The PZ also contains several other genes that encode proteins linked to pathogenesis, including the MAC/perforin domain protein and five phospholipase D (PLD) [30, 31]. The presence of two cytotoxic genes make the *C. pecorum* PZ unique compared to the PZ from other *Chlamydia* species. Although of similar size, the *tox*A and *tox*B genes share only 43% sequence identity on the amino acid level with several conserved motifs. The N-terminal regions of the *tox*A/B genes contain a catalytic glycosyltransferase domain. Interestingly, in the genome of *C. pecorum* MC/MarsBar, a SNP (C-T) in the *tox*B gene resulted in a premature stop codon at position 6440, truncating the gene. The presence of the premature stop codon was confirmed with PCR amplification and sequencing of the *tox*B gene in the *C. pecorum* MC/MarsBar strain, as well as the other two koala and a sheep strain. The *tox*B gene is only truncated in *C. pecorum* MC/MarsBar and full-length homologs are present in the other four *C. pecorum* genomes.

### The presence of a limited number of pseudogenes further highlights genetic variation between koala *C. pecorum*strains

In addition to *toxB*, another five pseudogenes were identified in the three koala *C. pecorum* genomes, providing additional evidence of genetic diversity between *C. pecorum* strains isolated from koalas (Table 3). The pseudogenes in *C. pecorum* were caused by premature stop codons within the gene sequences as a result of SNPs. *C. pecorum* IPTaLE has three pseudogenes, one being the *pyr*E gene (CpecA_0392) that codes for an orotate phosphoribosyltransferase. The functions of the other two pseudogenes in *C. pecorum* IPTaLE are unknown, although one of the genes (CpecA_0640) contains a signal peptide at the N-terminus suggesting that the encoded protein targets a secretory pathway. The genomes of *C. pecorum* MC/MarsBar and *C. pecorum* DBDeUG each have a single pseudogene that contains transmembrane domains; however their actual functions are unknown. All six pseudogenes, including *toxB*, identified in the koala *C. pecorum* genomes are intact in the all the livestock *C. pecorum* genomes.

**Table 3:** **Pseudogenes caused by premature stop codons in** ***C. pecorum*** **strains isolated from koalas**

Strain	Locus tag	Protein description	Percentage of gene truncated
*C. pecorum* IPTaLE	CpecA_0392	Orotate phosphoribosyltransferase	64%
	CpecA_0639	Hypothetical protein	38%
	CpecA_0640	Hypothetical protein	24%
*C. pecorum* MC/MarsBar	CpecG_0412	Hypothetical protein	19%
	CpecG_0814	Cytotoxin	37%
*C. pecorum* DBDeUG	CpecF_0874	Hypothetical protein	59%

### Some but not all predicted koala *C. pecorum*pseudogenes are widely distributed in *C. pecorum*strains detected from across the koala’s host range

**Figure 6:**
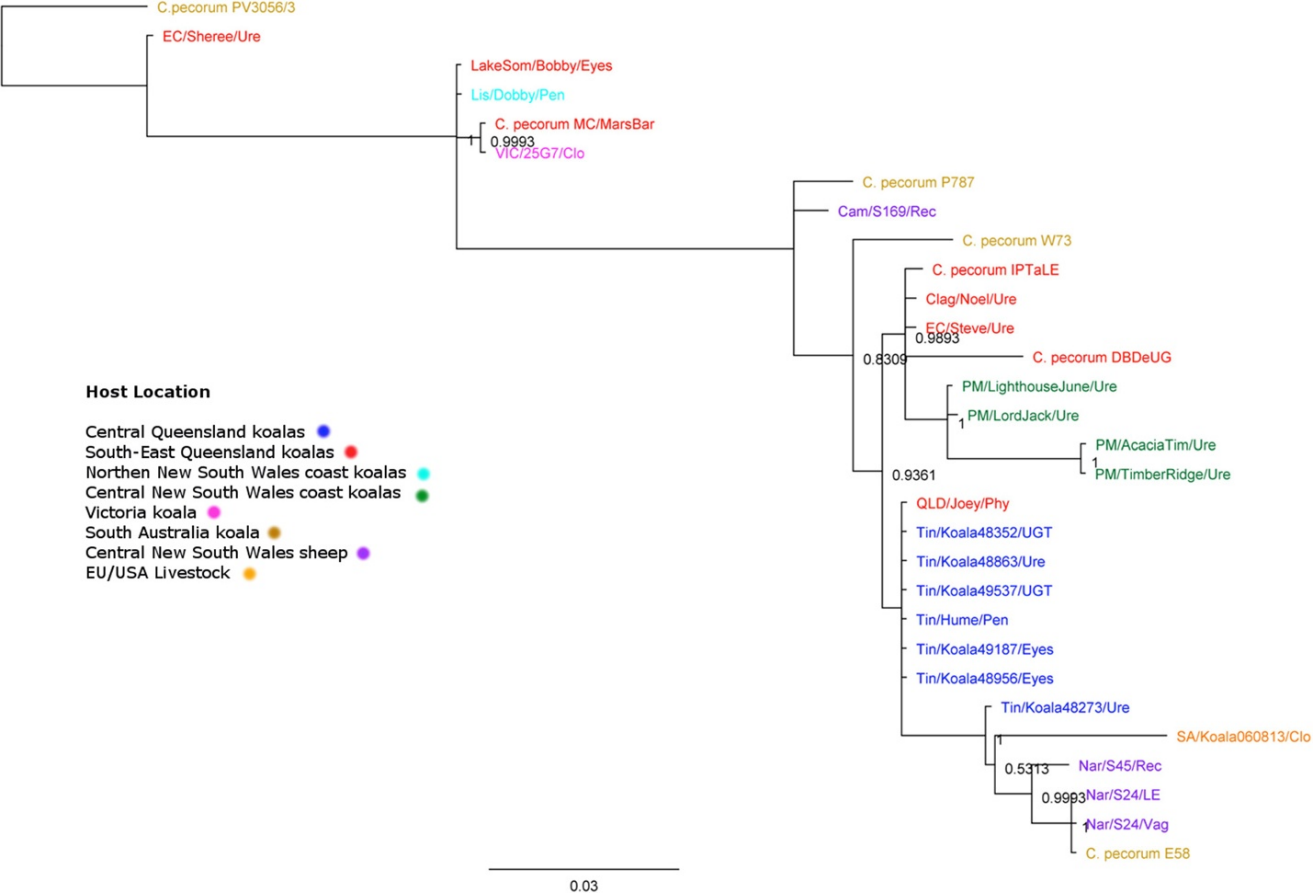
**Bayesian phylogenetic tree of concatenated sequence of five pseudogene fragments.** The tree was constructed using MrBayes HKY85 model based on the concatenated sequences of the five pseudogenes from 30 *C. pecorum* detected in koalas and livestock. Posterior probabilities > 0.85 are displayed on tree nodes. The colouring of the strain name as per the legend indicates the location of each strain. The following abbreviations denote anatomical site of the host where the swab sample was collected: Rec (Rectal), Jnt (Joint), LE (Left eye), Ure (Urethra), Pen (Penile), Vag (Vaginal) and Clo (Cloaca).

In a preliminary investigation into the broader genetic diversity of *C. pecorum* in Australian animals, we PCR amplified and sequenced partial regions of the six pseudogenes identified in the koala *C. pecorum* genomes from 73 *C. pecorum* PCR positive samples collected from the (a) conjunctival and urogenital tract sinuses of koalas from populations in Queensland, New South Wales, Victoria and South Australia (n = 65); and (b) conjunctiva and rectums of Australian sheep (n = 8) (see Additional file 4). The list of *C. pecorum* strains chosen was based on PCR-positive swab samples that have sufficient chlamydial DNA for multiple PCR amplification and to ensure an even distribution of strains from each Australian state. Out of the six pseudogenes identified from the genome sequences, four were also found to be pseudogenes in the clinical samples. The *toxB* gene was found to be truncated in some (n = 11) but not all of the koala *C. pecorum* positive samples from populations in South-East Queensland, South Australia and Victoria. Indeed, we were able to identify 11 unique *toxB* gene fragment sequences amongst the collection examined, with sequences varying in similarity between 84.3 – 99.7% across the livestock and koala samples (see Additional file 5). Similarly, one of the pseudogenes (CpecA_0641) from *C. pecorum* IPTaLE was also truncated in three urogenital *C. pecorum* positive swabs from three different koala hosts, however the truncation is caused by a different mutation than in *C. pecorum* IPTaLE. The pseudogene (CpecG_0412) from *C. pecorum* MC/MarsBar was found to be truncated in another two *C. pecorum* positive samples collected from the cloaca and urethra of a koala in Victoria and NSW, respectively. The pseudogene (CpecF_0874) from *C. pecorum* DBDeUG was also confirmed to be a pseudogene in seven *C. pecorum* positive samples from the conjunctiva and genital tracts of koalas in Queensland only. Interestingly, the remaining two pseudogenes (*pyrE* and CpecA_0640) from *C. pecorum* IPTaLE were intact in all koala *C. pecorum* samples analysed. Although only a smaller sample set was analyzed, the evidence that all pseudogenes identified and confirmed in the koala *C. pecorum* genomes and clinical samples appeared to be intact in Australian livestock was notable. While this observation appeared to further distinguish koala from livestock *C. pecorum* strains, it is also worth noting that a number of the new partial gene sequences identified in this analysis for each of these marker genes were otherwise identical (see Additional file 5). This was confirmed with a phylogenetic tree based on the concatenated sequences of five of the six pseudogenes – *pyrE* gene sequences were difficult to amplify for a number of samples – not showing any clear distinction between koala and livestock strains from Europe, the USA and Australia (Figure 6).

## Discussion

While the initial sequencing of chlamydial genomes has provided important insights into the general biology of the chlamydiae, reconciling the minor genetic differences between different strains of the same species with particular diseases, hosts and sites of infection has been more challenging. *C. pecorum* is a useful model to investigate adaptation to different niches since it is a major pathogen with a broad host range and tissue tropisms [11–13]. Like most *Chlamydia* species, *C. pecorum* genomes are conserved in terms of gene content and order [21]. This high degree of gene conservation is believed to be the result of *Chlamydia’*s intracellular lifestyle, which reduces exposure to frequent lateral gene transfer events that often cause phenotypic changes in other bacterial genera [32]. Therefore, small variations in the *C. pecorum* genomes are the likely causes for different phenotypic traits that are observed in different strains.

When it comes to investigating the genetic diversity of *C. pecorum,* one of the limiting factors has been the lack of genome sequences for *C. pecorum* strains isolated from different animals, in particular strains isolated from koalas. In this study we examined the draft genomes of three *C. pecorum* strains that were isolated from koalas in South-East Queensland, Australia. Each of the three koala *C. pecorum* genomes represents a different genotype, based on allelic differences in the *omp*A gene. Also sequenced, as part of this study, is the genome of *C. pecorum* VR629 (IPA), a strain that was isolated from the joint of a sheep suffering polyarthritis in the USA. The *C. pecorum* VR629 genome along with the four publically available *C. pecorum* genomes serve as references for comparison to the koala *C. pecorum* genomes, to assess the level of genetic diversity between *C. pecorum* strains [21, 24]. The PZ, the PMP gene cluster and the 77 kbp region near the origin of replication are hotspots for SNPs accumulation in the Australian koala strains and the European livestock strains. A similar high degree of SNPs is also observed in these same regions in *C. trachomatis* genomes (see Additional file 6). The gene content of the PZ and the 77 kbp region were also compared between *C. trachomatis* and *C. pecorum* using discontinuous megablast [33]. While the gene content of the PZ is significantly different between *C. pecorum* and *C. trachomatis,* the only difference in the 77 kbp hotspot region between both species is that the putative virulence genes in *C. pecorum* are absent in *C. trachomatis*. The PZ and the 77 kbp region are highly conserved between the two American *C. pecorum* strains but additional genome sequencing of isolates from the USA is needed to confirm if this level of conservation is unique to USA strains.

Identification of SNPs between *C. pecorum* genomes from livestock and koalas has provided insight into which genes are likely to be involved in host adaptation. The most rapidly evolving genes in *C. pecorum* are the genes coding for the PMPs, a family of proteins that may be important for adhesion of *Chlamydia* to host cells [4, 34, 35]. In particular, the *pmpG* subfamily represents the most variable class of PMPs in *C. pecorum*, which is also the most rapidly evolving PMP class in *C. psittaci* [6]. Despite the variation in nucleotide sequences, the members of the PMP family possess a conserved domain structure that includes the C-terminal autotransporter beta-domain, a central domain unique to this family of proteins and a N-terminal domain that is involved in adhesion [36]. PMPs share sequence similarity to the autotransporter class of proteins, which have a diverse array of functions and often play an essential role in pathogenesis, thus the observed variation could also contribute to immune evasion [37].

This study has identified several novel putative virulence genes that could be specifically involved with adaptation to different hosts. These virulence genes include a putative surface protein *Srp*A1 and two putative Type III effectors. Type III effectors are virulence factors often involved in interactions with host cellular proteins in order to enhance the survival of the bacteria [38]. Amino acid changes introduced by non-synonymous SNPs could alter the binding domains of the effectors, altering their affinity to cellular targets. The other potential virulence factor of interest is the putative surface protein SrpA1; the observed variation in the protein sequence could alter binding and/or recognition to surface receptors of host cells, however, experimental confirmation is obviously required for further investigations. However, the accumulation of non-synonymous SNPs in srpA1 and the two putative effector genes could also be due to a selective pressure for avoiding antibody and cellular immune responses.

Our comparison of the *C. pecorum* genomes revealed the presence of six pseudogenes in the strains isolated from koalas, one of these pseudogenes being the *toxB* gene located in the PZ in *C. pecorum* MC/Marsbar. *C. pecorum* typically possesses two full-length cytotoxin genes in the PZ, designated *tox*A and *tox*B. The full-length *Chlamydia* cytotoxin is similar to the large clostridial cytotoxin (LCTs) from *Clostridium difficile*, which also has the glycosyltransferase domain in the N-terminal region of the protein. This domain has been shown to interfere with eukaryotic cells by glycosylating GTP binding proteins of the Ras superfamily, inactivating them and leading to disassembly of the actin cytoskeleton [39, 40]. The LCTs also include a domain for binding to the surface receptors of eukaryotic cells and a transmembrane segment that is involved in translocation into the cytoplasm [41]. *C. pecorum* MC/MarsBar has a full-length *tox*A gene and a shorter *tox*B gene that has been truncated by a premature stop codon. The truncation of *tox*B was observed in 11 of the 65 *C. pecorum* positive swabs samples for which partial gene sequences could be amplified from wild koala, suggesting that it could be a recent mutation since the remaining sequences identified were otherwise intact. It is plausible that the C-terminal domain of the *tox*B gene is being deleted to reduce the energetic cost of maintaining the full-length gene [42].

Out of the other five pseudogenes in the koala *C. pecorum* genomes, only the *pyrE* gene has a predicted function. The gene *pyr*E encodes an orotate phosphoribosyltransferase, an enzyme that is part of the *de novo* pyrimidine biosynthesis pathway [43]. *Chlamydia* spp. cannot synthesise pyrimidine nucleotides *de novo* as key genes involved in this pathway are missing [44], but instead must rely on a salvaging pathway that involves transporting ribonucleotides directly from the host cell [45]. The presence of the *pyr*E gene could be a remnant from when *Chlamydia* was able to synthesise pyrimidine *de novo;* this is supported by the observed fragmentation of *pyr*E in *C. pecorum* IPTaLE, indicating gene decay. Interestingly, despite this potentially important observation, the *pyrE* gene was found to be intact in all koala *C. pecorum* strains that were screened, raising questions over whether this SNP may have resolved during laboratory passage. In addition to this pseudogene, there are two other pseudogenes that are unique to the *C. pecorum* IPTaLE conjunctival isolate, compared to the sequenced urogenital DBDeUG and MC/Marsbar strains. However, while one of these pseudogenes was found to be intact in all samples tested, the other (CpecA_0641) was confirmed to be a pseudogene in three koala urogenital samples from the same geographic region and therefore both of these genes are unlikely to be involved in tissue tropism.

Sequence alignments of the pseudogene fragments revealed that some genotypes are specific to *C. pecorum* detected in Australian livestock and koala hosts (see Additional file 5). However, a phylogenetic analysis of the concatenated pseudogene sequences showed that, collectively, there was no host specific separation in contrast to our whole genome phylogenies (Figure 6). The observation that (a) multiple *C. pecorum* genotypes can be circulating in a single population of animals; and (b) individual genotypes can be found across multiple populations of koalas or livestock is otherwise consistent with our previous fine-detailed molecular epidemiological investigations [15, 18, 19].

## Conclusions

While the primary focus of this study was to examine the genetic differences potentially associated with *C. pecorum* host adaptation, the observed accumulation of genetic changes in koala *C. pecorum* strains provides an opportunity to speculate on the origin of this pathogen in the koala. The koala *C. pecorum* genomes contain a small number of pseudogenes and gene truncations, which are otherwise intact in the livestock genomes. These mutations suggest that *C. pecorum* is evolving to adapt to the koala through the loss of genes that are no longer necessary. Although our genome phylogenies do not yet support this, the most parsimonious explanation for this observation is that koala *C. pecorum* strains have derived from livestock strains and are undergoing genetic changes to better adapt to the new host. Interestingly enough, if this is true, then it would be the reverse of what we have previously observed for koala *Chlamydia pneumoniae*, whereby the genome of the koala strain was found to be largely intact compared to human strains, suggesting that the koala strains were ancestral [44]. In order to validate that koala *C. pecorum* strains are indeed derived from livestock strains it is necessary to reconstruct the evolutionary history of *C. pecorum* using phylogenetic and molecular clock analyses. However, additional *C. pecorum* strains will need to be sequenced in order to fill in the missing links from this comparison including analysis of *C. pecorum* isolates from Australian livestock. Indeed, a limitation of the phylogenetic analysis performed in this study is the small number of available *C. pecorum* genomes. *C. pecorum* PV3056 was expected to cluster together with *C. pecorum* W37 and *C. pecorum* P787 based on the geographic location of these strains but the PV3056 strain is phylogenetically distinct from the other *C. pecorum* strains. Therefore, it is important to sequence more *C. pecorum* strains in order to fully understand the phylogenetic structure of this species but also to learn more about the role of the observed limited gene variation in disease pathogenesis and tissue and host adaptation.

## Methods

### Bacterial strains, chlamydial cell culturing and enrichment for genome sequencing

Three koala *C. pecorum* strains utilised for genome sequencing and comparative genomics were propagated in our laboratory. Swab samples were collected from three wild koalas residing in South East Queensland, Australia and stored in SPG transport media [46]. *C. pecorum* DBDeUG (QLD/SEF/UGT) strain was isolated from the urogenital tract of a wild female koala suffering from a urogenital tract infection, while the *C. pecorum* IPTaLE (QLD/IpsA/Eye) strain was isolated from the left conjunctiva of a wild male koala suffering from conjunctivitis. *C. pecorum* MC/MarsBar strain, previously described by Marsh *et al*. [19], was isolated from a female koala suffering from severe ocular and urogenital tract disease. The collection of these swabs by qualified veterinarians as a part of routine diagnostic testing and the subsequent *Chlamydia* culturing has been considered by the Queensland University of Technology (QUT) Animal Ethics Committee and approved as Tissue Use Notification # 1100000718. We also sequenced the genome of the ovine *C. pecorum* polyarthritis strain IPA (ATCC VR629), originally isolated from the joint fluid of a sheep in Iowa, USA [47]. In addition, we also made use of the full genome sequence of *C. pecorum* E58, an isolate that was collected from the brain of a calf suffering sporadic bovine encephalomyelitis [24]. Also included are the complete genomes of *C. pecorum* W73, *C. pecorum* P787 and *C. pecorum* PV3056, which were sequenced from strains infecting livestock animals in Europe [21].

The koala *C. pecorum* isolates were individually propagated in Hep-2 cells while the ovine *C. pecorum* isolate was propagated in McCoy cells. Following density gradient centrifugation, ultrapurified EBs for each strain were treated with DNAase and then purified using a QIAamp DNA Mini Kit (Qiagen), according to the manufacturer’s instructions, followed by repeated sodium acetate/ethanol precipitation and pellet resuspension in 0.1 M TE buffer.

### Prospective screening of koala samples using *C. pecorum-*specific PCR screen

To expand the analysis of *C. pecorum* strains from koalas outside of South-East Queensland, conventional PCR-based *C. pecorum* specific screening was performed on a range of koala samples collected from koala populations in Queensland, South Australia and New South Wales. From these regions, a total of 156 swabs were collected from 62 koalas presenting for treatment at the Australia Zoo Wildlife Hospital (n = 32), Adelaide Hills Animal Hospital (n = 23), and Port Macquarie Koala Hospital (n = 13), respectively. For each animal, sampling included a collection of conjunctival and urogenital sinus samples, while nasal samples were also collected from the South Australian koalas. In addition to the prospectively screened samples, a collection of 35 previously analysed *C. pecorum* positive samples were included in this analysis including samples from (i) various koala populations in South-East Queensland (n = 20), New South Wales (n = 6) and Victoria (n = 1); and (ii) Australian livestock sampled in Central New South Wales (n = 8) [18, 19]. Prospectively collected samples were screened for the presence of *C. pecorum* DNA using a *C. pecorum* specific qPCR assay, that targets a 202 base pair region of the *C. pecorum* 16S rRNA gene, as previously described [48] using extracted DNA as a template.

### Genome sequencing

The genomes of the four *C. pecorum* strains were sequenced using Illumina HiSeq to produce, paired-end 100 base-pair reads. Read quality was checked with FASTQC and filtering was performed on the reads with PrinSeq-Lite to ensure a mean base-pair quality of score greater than 20. The paired-end reads were randomly selected so that coverage of 100× was achieved for each genome. The genomes were assembled *de novo* using SOAPdenovo with an optimal *k-mer* of 33, which was determined by individually testing odd *k-mer* values ranging from 25 to 35. The genomes were further assembled into a single scaffold using GapCloser [49]. *C. pecorum* VR629 (ATCC IPA) was assembled into four contigs, while the genomes for the three koala *C. pecorum* strains (*C. pecorum* DBDeUG (QLD/SEF/UGT), *C. pecorum* IPTaLE (QLD/IpsA/Eye) and *C. pecorum* MC/MarsBar) were assembled into contigs ranging from 8 to 13 contigs. The contigs for each genome were ordered against the complete genome of *C. pecorum* E58 (accession number: CP002608) [24]. The average N50 contig size is 479,694 base pairs and the average size of the assembled genomes is 1.1 megabase pairs. The four draft *C. pecorum* genomes were automatically annotated using GenDB [50]. The genome sequences for the three koala strains and the sheep were deposited in Genbank under accession numbers AZBE01000000 for *C. pecorum* IPTaLE, AZBB01000000 for *C. pecorum* DBDeUG, AZBC01000000 for *C. pecorum* MC/Marsbar and AZBD01000000 for *C. pecorum* VR629.

### Phylogenetic analyses and genome comparison

The following genome sequences were used in comparative and phylogenetic analyses with the four *C. pecorum* genomes sequenced in this study: *C. muridarum* NIGG (accession number: AE002160), *C. trachomatis* A/HAR-13 (accession number: CP000051), *C. trachomatis* L2/434/Bu (accession number: AM884176), *C. caviae* (accession number: AE015925), *C. felis* Fe/C-56 (accession number: AP006861), *C. abortus* S26/3 (accession number: CR848038), *C. psittaci* 6BC (accession number: CP002549), *C. pneumoniae* AR39 (accession number: AE002161), *C. pneumoniae* LPCoLN (accession number: CP001713), *C. pecorum* E58 (accession number: CP002608), *C. pecorum* P787 (accession number: CP04035), *C. pecorum* W73 (accession number: CP004034) and *C. pecorum* PV3056/3 (accession number: CP04033).

A phylogenetic analysis was performed using 152 orthologous genes with > 70% nucleotide identity and < 20% difference in gene length from the 14 *Chlamydia* genomes, which includes representatives from all major species. The nucleotide sequences of the 152 genes were extracted from each genome and individually aligned using MUSCLE [51], and then concatenated. A phylogenetic tree was constructed by the maximum-likelihood method using the General Time Reversible (GTR) model with PhyML 3.0 [52]. Bootstrap values were calculated using 500 replicates.

Pairwise whole genome comparisons of the four draft *C. pecorum* genomes were performed using BLASTn and visualised with the Artemis Comparison Tool [53]. Figures of the whole genome comparison were generated using BRIG (BLAST Ring Image Generator) [54] and Easyfig [25]. Selection analysis was performed with KaKs Calculator 2.0 using the LWL model [29, 55]. The selective pressure acting on genes can be measured by calculating the ratio of non-synonymous to synonymous substitutions (d_n_/d_s_). Low d_n_/d_s_ values (less than 1) are indicative of purifying selection, which means that the gene is being maintained and most of the substitutions are synonymous. On the other hand, high d_n_/d_s_ ratios (greater than 1) are usually suggestive of positive selection in which the gene is accumulating non-synonymous substitutions that result in changes in the amino acid sequence of the encoded protein [56].

### SNP prediction

The filtered reads of each *C. pecorum* strain were mapped individually against the genome of *C. pecorum* E58 acquired with 12× coverage (Assembly ID: GCF_000204135.1) and the assembled scaffolds of the other *C. pecorum* genomes using the BWA-backtrack algorithm with BWA aligner [57]. The BWA parameters used include the number of differences allowed between the reference and query set at 0.04 and the number of differences allowed in the seed was 2. The maximum number of gaps allowed in the alignment was 1 and the gap penalty was set at 11. SNPs were predicted using the variant caller program, VarScan with default settings [58]. Progressive Mauve with default setting was used to align the complete *C. pecorum* genomes and call SNPs since the reads were not available [59]. A custom Perl script was used to determine if a SNP caused a synonymous change, non-synonymous change or a change that introduce premature stop codons within CDSs leading to the formation of pseudogenes. A custom R script was used to plot the distribution of SNPs across the genome with a window size of 100 kbp. Predicted SNPs within the PZ, PMP gene cluster and the 77 kbp hypervariable region as well as within the six potential pseudogenes were manually and visually inspected using the BAM files generated from the BWA read mapping in Artemis.

### Targeted PCR amplification of predicted *C. pecorum*pseudogenes in koala and livestock *C. pecorum*strains

In order to confirm the truncation of each pseudogene and to survey a broader selection of *C. pecorum* samples from koalas and Australian livestock, *C. pecorum* specific primers were designed for PCR amplification of a 250-500 bp region of each gene, which includes the predicted stop codons observed in our genome analyses (see Additional file 7). Each PCR assay was prepared to a total volume of 50 μl, consisting of 1 X Amplitaq Gold 360 Mastermix (Life Technologies, Victoria, AUS), 0.3 μM of Forward and Reverse primers (Sigma-Aldrich, New South Wales, AUS) and 3 μl DNA template, of an average concentration of 30 ng/μl. The cycling conditions for the PCR amplification of the six pseudogene fragments included initial denaturation (10 min, 95°C) followed by 40 cycles of denaturation (30s, 95°C), annealing (*tox*B 30s, 61.5°C; *pyr*E 30s, 56°C; C.pecA_0641 30s, 53°C; CpecA_0640 and CpecG_0412 30s, 49°C; and CpecF_0874 30s, 58.6°C) and extension (1 min, 72°C), followed by a final extension (7 min, 72°C). Negative controls (dH_2_0 and no template) were included in each amplification assay. Primer characteristics are outlined in Additional file 7.

Each PCR product was directly sequenced using a BigDye Terminator v3.1 Cycle Sequencing kit (Life Technologies, Victoria, Australia) and subsequently purified according to the manufacturer’s instructions. Sequencing was performed at the Queensland University of Technology DNA sequencing facilities, using the Applied Biosystems ABI3500 Gene analyser. The acquired sequences of the livestock and koala *C. pecorum* fragments for each gene were aligned using ClustalW [60] and translated as implemented in Geneious Pro 7, in order to confirm the presence of the observed stop codon in each of the strains analysed. A phylogenetic tree based on the concatenation of five pseudogene fragment sequences was constructed using MrBayes [61]. The nucleotide sequences for each of the six pseudogene fragments amplified in this study from the *C. pecorum* positive samples are available in GenBank under the accession numbers KJ804269-KJ804400.

## Electronic supplementary material

Additional file 1: **Whole genome comparison of** ***C. pecorum*** **IPTaLE.** The innermost ring shows GC content (black) and the second inner ring shows the read coverage (red). Genome regions with coverage more than one standard derivation from the mean coverage are represented as blue spikes. Contig boundaries are shown as alternating red and blue bars on the third ring. The remaining rings show the genomic similarity to the other seven *C. pecorum* genomes (*C. pecorum* MC/MarsBar, *C. pecorum* DBDeUG, *C. pecorum* VR629, *C. pecorum* E58, *C. pecorum* W73, *C. pecorum* P787 and *C. pecorum* PV3056) and the complete genomes of *C. pneumoniae* LPCoLN, *C. psittaci* 6BC and *C. caviae* GPIC. The green rings indicate the koala *C. pecorum* genomes and the blue rings represent the livestock *C. pecorum* genomes. BLASTn matches with an identity above 70% are coloured, while non-matching regions appear as blank spaces in the ring. The outer ring also marks the location of the plasticity zone, the tryptophan biosynthesis operon and a polymorphic gene cluster that encodes several membrane proteins. The image was prepared using BRIG [54]. (JPEG 1 MB)

Additional file 2: **List of genes in the 77-kbp SNP hotspot region.** A table of genes in the 77-kbp SNP hotspot regions from *C. pecorum* MC/MarsBar. The number of SNPs and ka/ks ratio is based on the comparison of these genes between *C. pecorum* MC/MarsBar and *C. pecorum* E58. (XLSX 38 KB)

Additional file 3: **Alignment of the surface anchor protein SrpA1 from** ***C. pecorum*** **.** The multiple protein alignment shows the full length of the SrpA1 surface protein from *C. pecorum* IPTaLE, *C. pecorum* MC/MarsBar, *C. pecorum* DBDeUG, *C. pecorum* VR629 and *C. pecorum* E58. The alignment is coloured using the BLOSUM62 scoring matrix. The C-terminal domain is conserved in all five *C. pecorum* genomes but the N-terminal part of the protein contains significant variation. (PDF 1 MB)

Additional file 4: **Summary table of PCR screening for pseudogenes in** ***C. pecorum*** **positive samples.** The table shows the status of the six pseudogenes in 65 *C. pecorum* positive swab samples from koalas and sheep and in the seven available *C. pecorum* genome sequences. (XLSX 94 KB)

Additional file 5: **Nucleotide alignment of pseudogenes fragments amplified with PCR.** Nucleotide alignments of the different alleles of the six pseudogenes fragments identified in a PCR screening of 65 *C. pecorum* strains. (DOCX 528 KB)

Additional file 6: **SNP distribution in** ***C. trachomatis*** **genomes.** The histograms show the number of SNPs in relation to the genomic positions between different *C. trachomatis* genomes with a window size of 100 kb. The top graph shows the SNP distribution between *C. trachomatis* D/UW-3/CX (accession number: AE001273) and *C. trachomatis* A/HAR-13 (accession number: CP00051). The bottom graph shows the SNP distribution between *C. trachomatis* D/UW-3/CX and *C. trachomatis* L2/434/Bu (accession number: AM884176). The red boxes mark the SNP hotspot regions that were also observed in *C. pecorum*. (PDF 49 KB)

Additional file 7: **Table of primers used in this study.** Contains the list of primers used to amplify the six pseudogenes. (XLSX 10 KB)
