# Supplementary figures and images for: Comparative genomics of koala, cattle and sheep strains of Chlamydia pecorum
Source: BMC Genomics. 2014 Aug 8;15(1):667. doi: 10.1186/1471-2164-15-667 (PMC4137089; doi:10.1186/1471-2164-15-667)

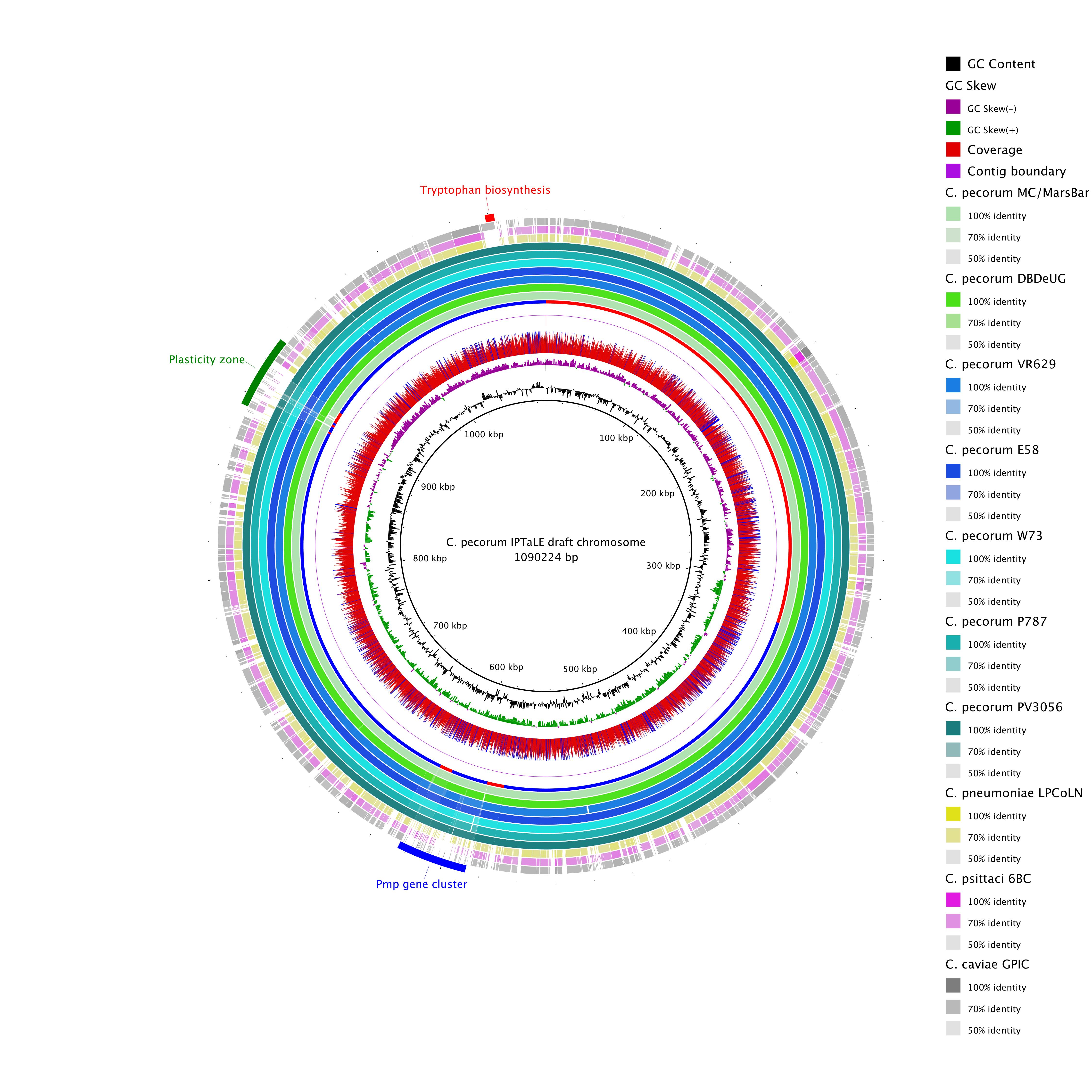

Supplement: Supplementary file 1 — Additional file 1: Whole genome comparison of C. pecorum IPTaLE. The innermost ring shows GC content (black) and the second inner ring shows the read coverage (red). Genome regions with coverage more than one standard derivation from the mean coverage are represented as blue spikes. Contig boundaries are shown as alternating red and blue bars on the third ring. The remaining rings show the genomic similarity to the other seven C. pecorum genomes (C. pecorum MC/MarsBar, C. pecorum DBDeUG, C. pecorum VR629, C. pecorum E58, C. pecorum W73, C. pecorum P787 and C. pecorum PV3056) and the complete genomes of C. pneumoniae LPCoLN, C. psittaci 6BC and C. caviae GPIC. The green rings indicate the koala C. pecorum genomes and the blue rings represent the livestock C. pecorum genomes. BLASTn matches with an identity above 70% are coloured, while non-matching regions appear as blank spaces in the ring. The outer ring also marks the location of the plasticity zone, the tryptophan biosynthesis operon and a polymorphic gene cluster that encodes several membrane proteins. The image was prepared using BRIG [54]. (JPEG 1 MB) [file 12864_2014_6356_MOESM1_ESM.jpeg]
