# Supplementary material for: Comparative genomics of koala, cattle and sheep strains of Chlamydia pecorum
Source: BMC Genomics. 2014 Aug 8;15(1):667. doi: 10.1186/1471-2164-15-667 (PMC4137089; doi:10.1186/1471-2164-15-667)
Supplement: Supplementary file 3 — Additional file 3: Alignment of the surface anchor protein SrpA1 from C. pecorum . The multiple protein alignment shows the full length of the SrpA1 surface protein from C. pecorum IPTaLE, C. pecorum MC/MarsBar, C. pecorum DBDeUG, C. pecorum VR629 and C. pecorum E58. The alignment is coloured using the BLOSUM62 scoring matrix. The C-terminal domain is conserved in all five C. pecorum genomes but the N-terminal part of the protein contains significant variation. (PDF 1 MB) [file 12864_2014_6356_MOESM3_ESM.pdf]

```
IPTaLE_SrpA1/1-634      1 MVNPLGTNEHEQIPIEPLASKDASSASSTEAASKTQEKKTEGPTPQAVERWSFLSAARNALSSLVNRLLGVASSAPTT---------------PTD 81
MC/MarsBar_SrpA1/1-634  1 MVNPLGTNEHEQIPIEPLASKDASSASSTEAASKTQEKKTEGPTPQAVERWSFLSAARNALSSLVNRLLGVASSAPTT---------------PTD 81
DBDeUG_SrpA1/1-646      1 MVNPLGTNEHEQIPIEPLASKDASSASSTEAASKTQEKKTEGPTPQAVERWSFLSAARNALSSLVNRLLGVASSTPTTSPDT-PSVDSTDPNPTD 95
VR629_SrpA1/1-651       1 MVNPLGTNEHEQIPIEPLASKDASSASSTEAASKTQEKKTEGPTPQAVETWSFLSAARNALSSLVNRLLGVASSTPTTSPDTSPSVDSTDPNPTD 96
E58_SrpA1/1-651         1 MVNPLGTNEHEQIPIEPLASKDASSASSTEAASKTQEKKTEGPTPQAVETWSFLSAARNALSSLVNRLLGVASSTPTTSPDTSPSVDSTDPNPTD 96

IPTaLE_SrpA1/1-634     82 PVDPTPPTPTFDDYKTQAETAYNTFLTSTDYSAVQAAAVSLQEAVNKMNELAAEDTATEEQKTTAAEWNTKNTNVTQVNAQLTEVSQLLQEIENLG 177
MC/MarsBar_SrpA1/1-634 82 PVDPTPPTPTFDDYKTQAETAYNTFLTSTDYSAVQAAAVSLQEAVNKMNELAAEDTATEEQKTTAAEWNTKNTNVTQVNAQLTEVSQLLQEIENLG 177
DBDeUG_SrpA1/1-646     96 PVDPTPPPPTFDDYKTQAETAYNTFLTSTDYSEVQAAVTSLQAAVNKMNELATDD----QQRATATEWGEKNTKVTQVYADLEEISRLLEENKQYQ 187
VR629_SrpA1/1-651      97 PVDPTPPPPTFDDYKTQAETAYNTFLTSTDYSAVQAAAVSLQEAVNKMNELAAEDTATEEQKTTAAEWGEKNTKVTQVYADLEEISRLLEENKQYQ 192
E58_SrpA1/1-651        97 PVDPTPPPPTFDDYKTQAETAYNTFLTSTDYSAVQAAAVSLQEAVNKMNELAAEDTATEEQKTTAAEWGEKNTKVTQVYADLEEISRLLEENKQYQ 192

IPTaLE_SrpA1/1-634    178 SSASSLDTIELVQAAQLNSQEKAAQATEKLEAMETEENPPLGQVPASVQTLKERIEAANKNTETVTQAIIDAYNAGENAYGSVQQAMANNSQANID 273
MC/MarsBar_SrpA1/1-634 178 SSASSLDTIELVQAAQLNSQEKAAQATEKLEAMETEENPPLGQVPASVQTLKERIEAANKNTETVTQAIIDAYNAGENAYGSVQQAMANNSQANID 273
DBDeUG_SrpA1/1-646    188 SSASSLANIDLVNAALLKSEENAAAVEAKLDEMNAEESPPLGRVPASAEALKTQMDTVTEETKAITKVLIDAYNAGEDAYGSVEQAKANNSQANID 283
VR629_SrpA1/1-651     193 SSASSLANIDLVNAALLKSEENAAAVEAKLDEMNAEESPPLGRVPASAEALKTQMDTVTEETKAITKVLIDAYNAGEDAYGSVQQAMANNSQANID 288
E58_SrpA1/1-651       193 SSASSLANIDLVNAALLKSEENAAAVEAKLDEMNAEESPPLGRVPASAEALKTQMDTVTEETKAITKVLIDAYNAGEDAYGSVQQAMANNSQANID 288

IPTaLE_SrpA1/1-634    274 AANAQITQAKGTIQNALLTYPNSPVLQTALSRVTEAEAALQNIKPAEAPSSGGAASAGSAQHRSFSVGNVRVALLLDDAESESTATVLSGLRKMIE 369
MC/MarsBar_SrpA1/1-634 274 AANAQITQAKGTIQNALLTYPNSPVLQTALSRVTEAEAALQNIKPAEAPSSGGAASAGSAQHRSFSVGNVRVALLLDDAESESTATVLSGLRKMIE 369
DBDeUG_SrpA1/1-646    284 AANAQITQAKETIRNALQTYPNSPVLQTALSRVTEAEAALQNIKPAEAPSSGGAASAGSAQHRSFSVGNVRVALLLDDAESESTATVLSGLRKMIE 379
VR629_SrpA1/1-651     289 AANAQITQAKETIQNALQTYPNSPVLQTALSRVTAAEAALQNIKPAEAPSSGGAASAGSAQHRSFSVGNVRVALLLDDAESESTATVLSGLRKMIE 384
E58_SrpA1/1-651       289 AANAQITQAKETIQNALQTYPNSPVLQTALSRVTAAEAALQNIKPAEAPSSGGAASAGSAQHRSFSVGNVRVALLLDDAESESTATVLSGLRKMIE 384

IPTaLE_SrpA1/1-634    370 LFQSGTTVGGEAQKAETEAEA--ANDSATTEEAAQAAVDEALQTMAEAKTREGLINAMGQIASAAMVSAGVPQAAAAPVAKSVKQLYSASSSTSSK 463
MC/MarsBar_SrpA1/1-634 370 LFQSGTTVGGEAQKAETEAEA--ANDSATTEEAAQAAVDEALQTMAEAKTREGLINAMGQIASAAMVSAGVPQAAAAPVAKSVKQLYSASSSTSSK 463
DBDeUG_SrpA1/1-646    380 LFQSGTTVGGEAQKAETEAEAVAANDSATTEEEAQAAVEEALQTMAEAKTREGLINAMGQIASAAMVSAGVPQAAAAPVAKSVKQLYSASSSTSSK 475
VR629_SrpA1/1-651     385 LFQSGTTVGGEAQKAETEAEAVAANDSATTEEEAQAAVEEALQTMAEAKTREGLIDAMGQIASAAMVSAGVPQAAAAPVAKSVKQLYSASSSTSSK 480
E58_SrpA1/1-651       385 LFQSGTTVGGEAQKAETEAEAVAANDSATTEEEAQAAVEEALQTMAEAKTREGLIDAMGQIASAAMVSAGVPQAAAAPVAKSVKQLYSASSSTSSK 480

IPTaLE_SrpA1/1-634    464 RFADGYSAYQSLTDTLSSINDRGRNALDAATQSALSQPVPRRTETRARGSEDLSQRVARSIAEDSKTYGDIYARVGALESLSTVLQNNPNANDAEI 559
MC/MarsBar_SrpA1/1-634 464 RFADGYSAYQSLTDTLSSINDRGRNALDAATQSALSQPVPRRTETRARGSEDLSQRVARSIAEDSKTYGDIYARVGALESLSTVLQNNPNANDAEI 559
DBDeUG_SrpA1/1-646    476 RFADGYSAYKSLTDTLSSINDRGRNALDAATQSALSQPVPRRTETRARGSEDLSQRVARSIAEDSKTYGDIYARVGALESLSTVLQNNPNANDAEI 571
VR629_SrpA1/1-651     481 RFADGYSAYQSLTDTLSSINDRGRNALDAATQSALSQPVPRRTETRARGSEDLSQRVARSIAEDSKTYGDIYARVGALESLSTVLQNNPNANDAEI 576
E58_SrpA1/1-651       481 RFADGYSAYQSLTDTLSSINDRGRNALDAATQSALSQPVPRRTETRARGSEDLSQRVARSIAEDSKTYGDIYARVGALESLSTVLQNNPNANDAEI 576

IPTaLE_SrpA1/1-634    560 KQALMSEVNKPPKFGYPYVQLPSESMQKYLGKLMQEFVDGTKAAAERKMLSFDTRTQFIQQVLVNLGSLYAAYLS 634
MC/MarsBar_SrpA1/1-634 560 KQALMSEVNKPPKFGYPYVQLPSESMQKYLGKLMQEFVDGTKAAAERKMLSFDTRTQFIQQVLVNLGSLYAAYLS 634
DBDeUG_SrpA1/1-646    572 KQALMSEVNKPPKFGYPYVQLPSESMQKYLGKLMQEFVDGTKAAAERKMLSFDTRTQFIQQVLVNLGSLYAAYLS 646
VR629_SrpA1/1-651     577 KQALMSEVNKPPKFGYPYVQLPSESMQKYLGKLMQEFVDGTKAAAERKMLSFDTRTQFIQQVLVNLGSLYAAYLS 651
E58_SrpA1/1-651       577 KQALMSEVNKPPKFGYPYVQLPSESMQKYLGKLMQEFVDGTKAAAERKMLSFDTRTQFIQQVLVNLGSLYAAYLS 651
```
