# Supplementary material for: Comparative genomics of koala, cattle and sheep strains of Chlamydia pecorum
Source: BMC Genomics. 2014 Aug 8;15(1):667. doi: 10.1186/1471-2164-15-667 (PMC4137089; doi:10.1186/1471-2164-15-667)
Supplement: Supplementary file 5 — Additional file 5: Nucleotide alignment of pseudogenes fragments amplified with PCR. Nucleotide alignments of the different alleles of the six pseudogenes fragments identified in a PCR screening of 65 C. pecorum strains. (DOCX 528 KB) [file 12864_2014_6356_MOESM5_ESM.docx]

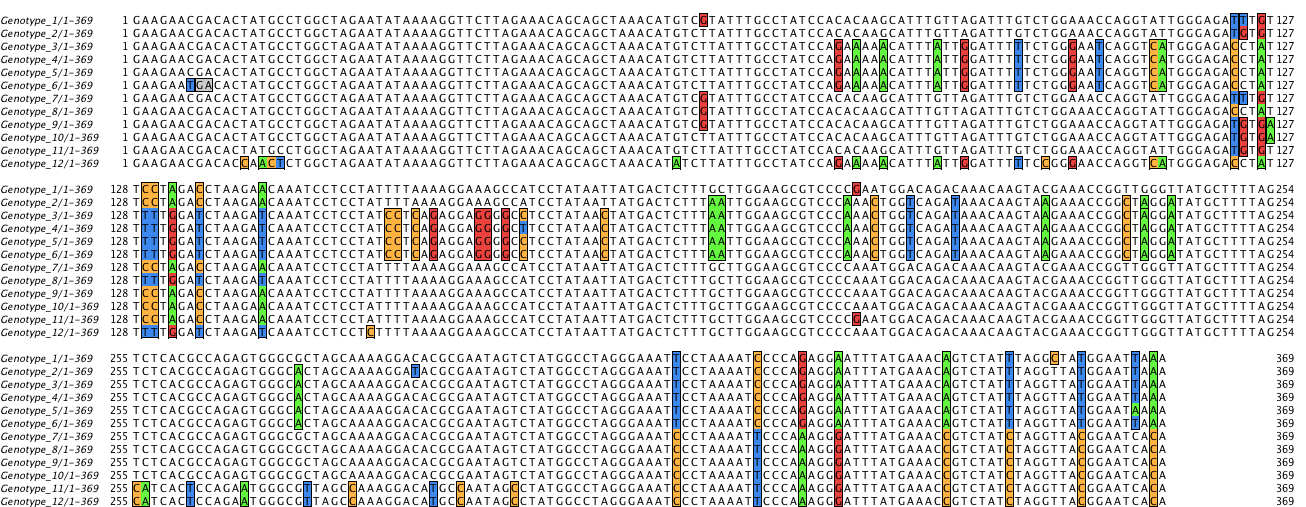


Figure S1: Sequence alignment of a 369 bp *C. pecorum toxB* fragment of the 12 *toxB* genotypes identified in this study. The alignment was constructed using Geneious Pro 7.1.3 with ClustalW. Base 1 of the sequence alignment corresponds with base 7699 in the complete *toxB* gene of the *C. pecorum* E58 genome sequence as the three *C. pecorum* koala strains are incomplete. Only one SNP causes a stop-codon (indicated by grey shading) at the third amino acid in genotype 6, which results in the truncation of the fragment.


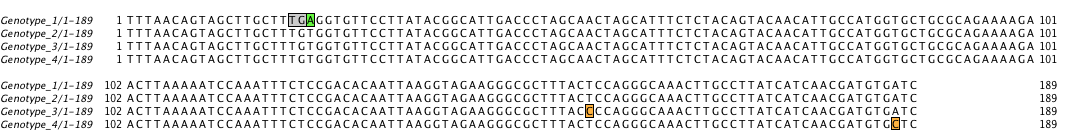


Figure S2: The sequence alignment of a 189 bp *C. pecorum* *pyrE* fragment identified 4 genotypes. The alignment was constructed using Geneious Pro 7.13 with ClustalW. Base 1 of the sequence fragment corresponds with base 217 in the complete *pyrE* gene of *C. pecorum* IPTaLE genome. Only one SNP causing a stop-codon was identified at the seventh amino acid in genotype 1 (indicated by grey shading), which results in the truncation of the gene.


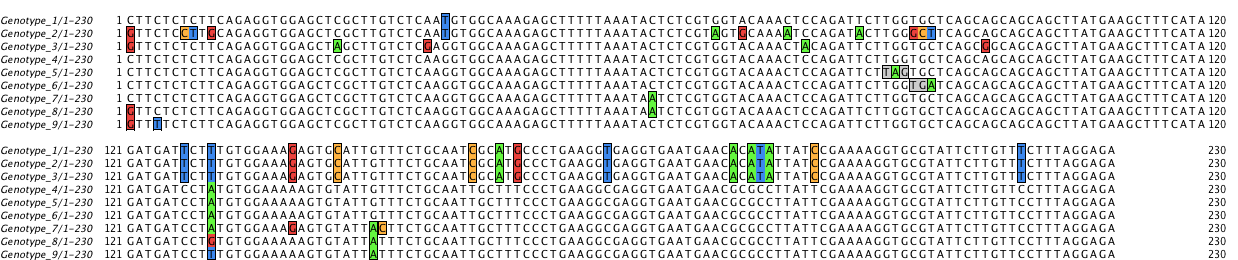


Figure S3: The sequence alignment of a 189 bp Cpec_0641 fragment identified 9 genotypes. The alignment was constructed using Geneious Pro 7.13 with ClustalW. Base 1 of the sequence fragment corresponds with base 760 in the complete hypothetical protein in CpecA_0641 gene. Two SNPs were identified to cause a stop-codon 29th amino acid in genotype 5 and the 30th amino acid in genotype 6 (indicated by grey shading), resulting in the truncation of the gene.


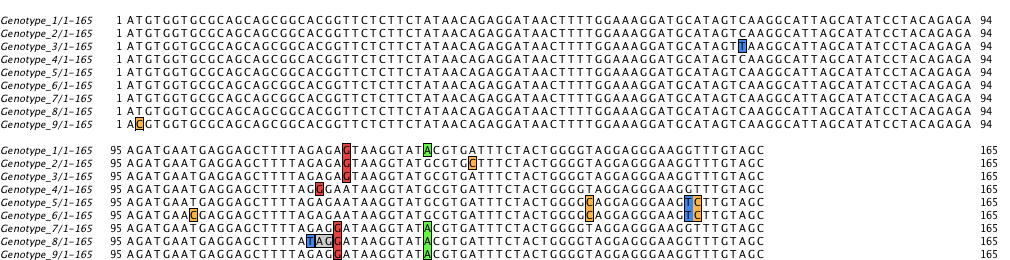


Figure S4: The sequence alignment of a 189 bp Cpec_0640 fragment identified 9 genotypes. The alignment was constructed using Geneious Pro 7.13 with ClustalW. Base 1 of the sequence fragment corresponds with base 760 in the complete hypothetical protein in CpecA_0640 gene. Only one SNP causing a stop-codon was identified at the 39th amino acid (indicated by grey shading) in genotype 8, which results in the truncation of the gene.

**
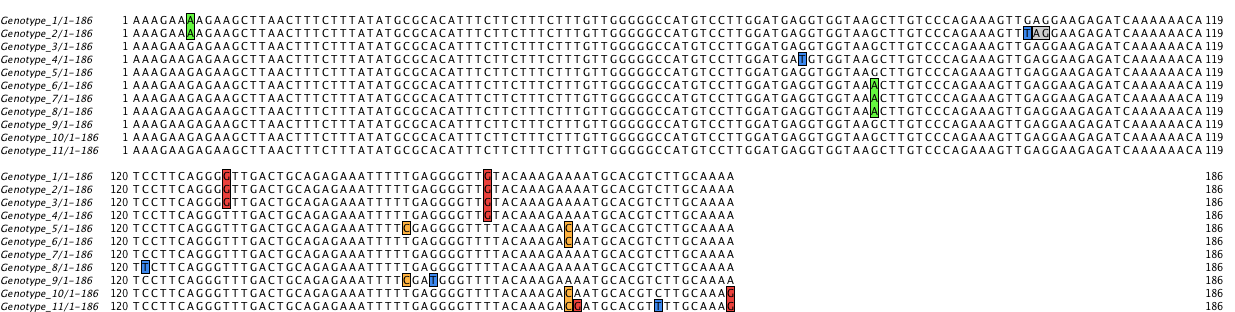
**

Figure S5: The sequence alignment of a 196 bp Cpec_0412 fragment identified 11 genotypes. The alignment was constructed using Geneious Pro 7.13 with ClustalW. Base 1 of the sequence fragment corresponds with base 2265 in the complete hypothetical protein in CpecG_0412 gene. Only one SNP causing a stop-codon was identified at the 34th amino acid in genotype 2 (indicated by grey shading), which results in the truncation of the gene.


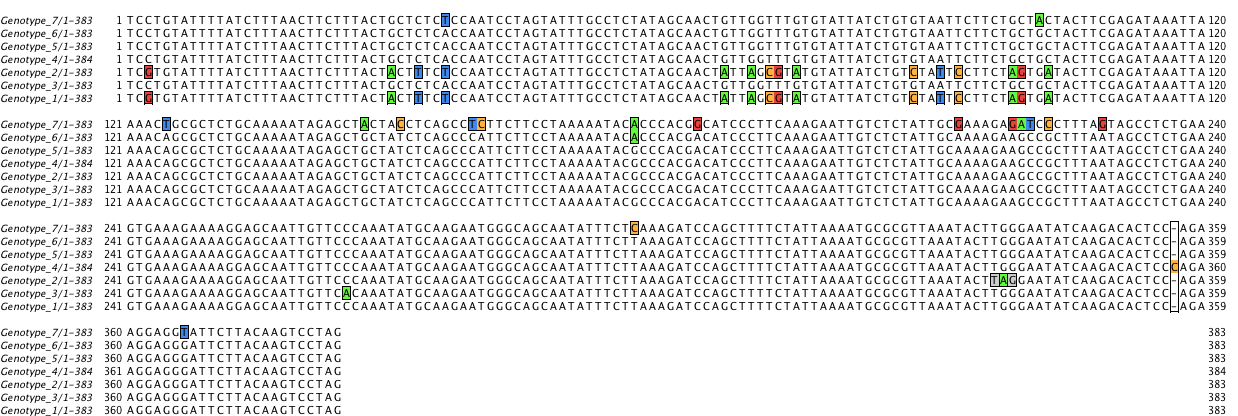


Figure S6: The sequence alignment of a 384 bp region of Cpec_0874 a total of 7 genotypes was identified. The alignment was constructed using Geneious Pro 7.1.3 with ClustalW. A single SNP was observed to cause a stop-codon at the 113^th^ amino acid in genotype 2 (indicated by grey shading) which results in the truncation of the fragment. Furthermore a single nucleotide insertion at position 357 resulted in a change in amino acid reading frame with the outcome of a second stop-codon in genotype 4. Position 1 indicates to the 136 base pair in the fulllength gene.
