# Supplementary material for: Comparative genomics of koala, cattle and sheep strains of Chlamydia pecorum
Source: BMC Genomics. 2014 Aug 8;15(1):667. doi: 10.1186/1471-2164-15-667 (PMC4137089; doi:10.1186/1471-2164-15-667)
Supplement: Supplementary file 6 — Additional file 6: SNP distribution in C. trachomatis genomes. The histograms show the number of SNPs in relation to the genomic positions between different C. trachomatis genomes with a window size of 100 kb. The top graph shows the SNP distribution between C. trachomatis D/UW-3/CX (accession number: AE001273) and C. trachomatis A/HAR-13 (accession number: CP00051). The bottom graph shows the SNP distribution between C. trachomatis D/UW-3/CX and C. trachomatis L2/434/Bu (accession number: AM884176). The red boxes mark the SNP hotspot regions that were also observed in C. pecorum. (PDF 49 KB) [file 12864_2014_6356_MOESM6_ESM.pdf]

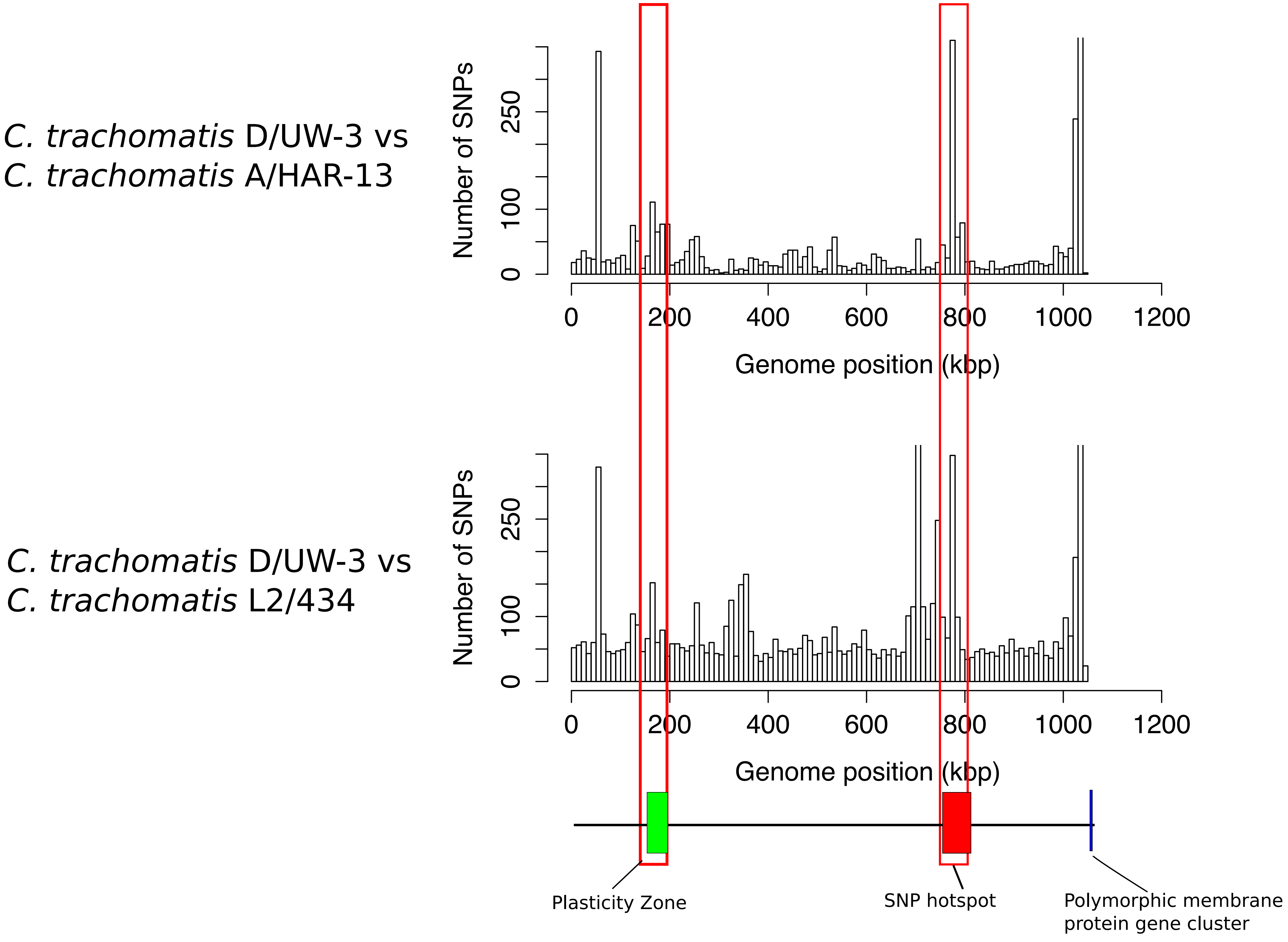
C. trachomatis D/UW-3 vs
C. trachomatis A/HAR-13
Number of SNPs
0
100
250
0
200
400
600
800
1000
1200
Genome position (kbp)
C. trachomatis D/UW-3 vs
C. trachomatis L2/434
Number of SNPs
0
100
250
0
200
400
600
800
1000
1200
Genome position (kbp)
Plasticity Zone
SNP hotspot
Polymorphic membrane
protein gene cluster
